# Predicting heterosis for egg production traits in crossbred offspring of individual White Leghorn sires using genome-wide SNP data

**DOI:** 10.1186/s12711-015-0088-6

**Published:** 2015-04-03

**Authors:** Esinam N Amuzu-Aweh, Henk Bovenhuis, Dirk-Jan de Koning, Piter Bijma

**Affiliations:** Animal Breeding and Genomics Centre, Wageningen University, Wageningen, the Netherlands; Department of Animal Breeding and Genetics, Swedish University of Agricultural Sciences, Uppsala, Sweden

## Abstract

**Background:**

The development of a reliable method to predict heterosis would greatly improve the efficiency of commercial crossbreeding schemes. Extending heterosis prediction from the line level to the individual sire level would take advantage of variation between sires from the same pure line, and further increase the use of heterosis in crossbreeding schemes. We aimed at deriving the theoretical expectation for heterosis due to dominance in the crossbred offspring of individual sires, and investigating how much extra variance in heterosis can be explained by predicting heterosis at the individual sire level rather than at the line level. We used 53 421 SNP (single nucleotide polymorphism) genotypes of 3427 White Leghorn sires, allele frequencies of six White Leghorn dam-lines and cage-based records on egg number and egg weight of ~210 000 crossbred hens.

**Results:**

We derived the expected heterosis for the offspring of individual sires as the between- and within-line genome-wide heterozygosity excess in the offspring of a sire relative to the mean heterozygosity of the pure lines. Next, we predicted heterosis by regressing offspring performance on the heterozygosity excess. Predicted heterosis ranged from 7.6 to 16.7 for egg number, and from 1.1 to 2.3 grams for egg weight. Between-line differences accounted for 99.0% of the total variance in predicted heterosis, while within-line differences among sires accounted for 0.7%.

**Conclusions:**

We show that it is possible to predict heterosis at the sire level, thus to distinguish between sires within the same pure line with offspring that show different levels of heterosis. However, based on our data, variation in genome-wide predicted heterosis between sires from the same pure line was small; most differences were observed between lines. We hypothesise that this method may work better if predictions are based on SNPs with identified dominance effects.

## Background

Commercial breeding programs for laying hens use crossbreeding schemes to exploit heterosis. The development of a reliable method to predict heterosis would greatly improve the efficiency of these breeding schemes by reducing their dependency on time-consuming and expensive field-tests of multiple pure-line combinations. Using egg production records from White Leghorn crosses, Amuzu-Aweh *et al.* [1] showed that heterosis can be predicted using the genome-wide average squared difference in allele frequency (SDAF) between the two parental lines, with an accuracy of ~0.5. With this method, one can predict which sire- and dam-line combinations have the highest potential for heterosis, and thus pre-select which crosses should be field-tested. However, the sires and dams within a pure line can be genetically different, and thus may vary in the heterosis that their offspring will express. In this study, genetic variation within the pure lines is quantified by the within-line heritabilities of target traits, which are in the same range as those reported in the literature [2,3], and by the expected heterozygosity within the lines. Exploring this individual variation is of interest to understand the genetic basis of heterosis, and also to increase the performance of commercial crossbred animals. In commercial animal breeding, selection intensity is highest for males, thus there may be possibilities to further exploit heterosis by selecting certain sires that are better suited for mating to a particular dam-line than others.

To this end, the aims of our study were to derive the theoretical expectation for heterosis due to dominance in the crossbred offspring of individual sires, and to investigate how much extra variance in heterosis can be explained by predicting heterosis at the individual sire level, rather than at the line level. We used genotypic data from 53 421 SNPs on 3427 individual White Leghorn sires, allele frequencies of six White Leghorn dam-lines, and phenotypic records on egg number and egg weight from 16 crosses between those lines, representing ~210 000 individual crossbred hens.

## Methods

### Population structure

Phenotypic records of ~210 000 crossbred hens that originated from nine purebred White Leghorn layer lines (three sire-lines and six dam-lines) were obtained from the Institut de Sélection Animale (ISA) B.V. These data are a subset of the population of chickens described in [1] since only records of crossbred hens for which sires had been genotyped were retained here. Following [1], sire-lines were coded as S1, S4 and S5, and dam-lines were D1, D2, D3, D4, D5 and D6. A cross produced by an S1 sire and a D1 dam is referred to as S1*D1 and its reciprocal as D1*S1. The D1 line was the only dam-line that was also used as a sire of crossbreds. The mating design produced 16 crosses (Table 1). Each of the 3427 sires was mated to one dam-line only, but to several hens of that particular line. Mate allocation was random, i.e. hens were artificially inseminated following the cage rows (personal communication, Jeroen Visscher, ISA, Hendrix). Pedigree on the dam side was not recorded.

**Table 1:** **Numbers of sires and records and mean egg number and weight for each cross**

**Cross**	**Number of genotyped sires**	**Number of crossbred progeny records** ^**1**^	**Average egg number**	**Average egg weight (g)**
D1*D4	301	2972	341.9	62.1
D1*S1	471	4808	342.6	60.7
S1*D1	259	3020	338.2	62.1
S1*D2	318	3768	339.0	60.2
S1*D3	243	3013	340.6	59.9
S1*D4	267	2921	334.1	60.9
S4*D1	48	340	331.3	62.5
S4*D2	43	318	336.2	61.1
S4*D3	16	201	336.9	60.4
S4*D5	366	3442	324.5	61.1
S4*D6	367	3588	326.1	60.0
S5*D1	33	285	345.1	62.4
S5*D2	40	353	343.1	60.9
S5*D3	42	354	345.2	60.8
S5*D5	308	2742	334.5	62.9
S5*D6	305	2674	332.9	61.1

^1^Each record is a cage-based average. There were ~ six hens per cage.

### Phenotypic data

The traits studied were egg number and egg weight. Phenotypic data were from routine performance tests for a commercial crossbreeding program, and were collected on test farms in the Netherlands, Canada and France from 2005 through 2010. Crossbred hens were beak-trimmed and housed in group-cages, and phenotypes were recorded per cage. A cage-based record is the mean record of all individuals within a cage. The number of cage-based records on egg number and egg weight per sire ranged from 1 to 23, with an average of ~11 cage-based records per sire, and about six hens per cage. Phenotypic data on pure lines was not used.

#### Egg number

Egg number is a cage-based record of eggs produced from 100 through 504 days of age calculated on a hen-day basis. Hen-day egg number was calculated as the total number of eggs laid in the cage divided by the total number of days that a hen was present in the cage (days are summed for all hens that were placed in the cage), and then multiplied by the maximum number of days the production period lasted. A full description of this trait and data editing criteria are in [1]. There were 34 799 cage-based records of egg number (Table 1).

#### Egg weight

Egg weight was measured five times throughout the production period: at around 25, 35, 45, 60 and 75 weeks of age. For each cage, the average weight of all eggs laid on a particular day was recorded. At the end of the production period, these five average weights were again averaged to give one value for egg weight per cage for the entire production period. There were 26 034 records of egg weight (Table 1).

### Genotypic data

Two types of genotypic information were used: individual 60K SNP genotypes of 3427 sires (1087 S1, 840 S4, 728 S5 and 772 D1), and allele frequencies of all nine pure lines in our data. The allele frequencies of the lines used only as dams (D2, D3, D4, D5 and D6) were obtained from pooled blood samples of 75 randomly selected males. For the lines used as sires (S1, S4, S5 and D1), we calculated the line allele frequencies from the individual sire genotypes. The same SNP array, the Illumina chicken 60K SNP BeadChip [4], was used for all genotyping. SNPs from the sex (Z) chromosome were excluded because females are the heterogametic sex in chickens (ZW), thus the sex chromosomes do not contribute to heterosis by dominance in females. We also excluded SNPs with a call rate less than 95% (161 SNPs). This brought the total number of SNPs used in this study to 53 421. Further details of the quality control criteria are in [1].

### Statistical analyses

#### Theory

At the line level, heterosis due to dominance is proportional to the squared difference in allele frequency between the two parental lines that produce a crossbred:

$$ \mathrm{Heterosi}{\mathrm{s}}_{ij}\kern0.5em =\kern0.5em {\displaystyle \sum_l{d}_l\;{\left({p}_{i,l}-{p}_{j,l}\right)}^2}, $$

where *d*_*l*_ is the deviation of the genotypic value of the heterozygote from the average of both homozygotes at locus *l*, *p*_*i*,*l*_ is the frequency of a particular allele at the bi-allelic locus *l* in parental line *i*, and *p*_*j*,*l*_ is the frequency of the same allele at locus *l* in parental line *j* [5]. Under the assumptions that (i) heterosis is due to dominance and (ii) the dominance deviation (*d*_*l*_) at a locus is independent of the squared difference in allele frequency between parental lines at that locus, when the phenotype of crossbred individuals is regressed on the mean squared difference in allele frequency between the two parental lines:

$$ {y}_{ijk}= sire\_lin{e}_i+ dam\_lin{e}_j+\beta \cdot \frac{1}{n_{loci}}{\displaystyle \sum_l{\left({p}_{i,l}-{p}_{j,l}\right)}^2}+{e}_{ijk}, $$

then the estimated partial regression coefficient is an estimator of the sum of dominance deviations over all loci, $\widehat{\beta}=Est.\left({\displaystyle \sum_ld}\right)$ [1,5] (note that the dominance deviations at all loci do not have to be equal for this statement to be true). This result holds even when phenotypic data on the pure lines is not available, as shown in detail in [1].

Thus, at the line level, heterosis due to dominance can be estimated using regression on the mean squared difference in allele frequency between parental lines. However, our aim was to predict heterosis at the sire level. For each sire, we calculated the allele frequency at each SNP locus. For example, for a SNP with alleles *a* and *A*, a sire can either be *aa, aA* or *AA*. If the population allele frequencies are expressed as *freq(A)*, then a sire’s allele frequency is simply the number of *A* alleles for that sire (0, 1 or 2) divided by the total number of alleles for a sire (which is 2). Thus, the allele frequencies for sires, corresponding to the three genotypes, are 0, 0.5 and 1. At first glance, to estimate ${\displaystyle \sum_ld}$, one might expect that regression can be done on the squared difference in the sire allele frequency and the allele frequency of the dam-line, using $1/{n}_{loci}\;{\displaystyle \sum_l{\left({p}_{s_i,l}-{p}_{j,l}\right)}^2}$, where ${p}_{s_i}$ is the allele frequency of the *s*^*th*^ sire from line *i,* and *p*_*j*_ is the allele frequency in the dam-line. This is, however, incorrect. Instead, we need to derive a term that is proportional to the expected heterosis due to dominance for crossbred offspring of a particular sire, say *s*_*i*_, from sire-line *i* that is mated to randomly chosen dams from dam-line *j.* In other words, we need to identify a term ${x}_{s_i,j}$, such that fitting a regression $\beta\;{x}_{s_i,j}$ yields a $\widehat{\beta}$ that is an estimator of ${\displaystyle \sum_ld}$.

In the following model:

1 $$ {y}_{s_ij}= sire\_lin{e}_i + dam\_lin{e}_j+\beta\;{x}_{s_i,j}+{e}_{s_ij}, $$

${y}_{s_ij}$ is the phenotypic record of an offspring of sire *s*_*i*_ from pure-line *i* mated to randomly chosen dams from pure-line *j, β* is a regression coefficient and ${x}_{s_i,j}$ is derived such that *β* becomes an estimate of ${\displaystyle \sum_ld}$.

The mean heterozygosity of pure-lines *i* and *j* is:

$$ \begin{array}{l}{\overline{H}}_{ii,\;jj}=\kern1em \frac{2{p}_i\left(1-{p}_i\right)+\kern0.5em 2{p}_j\left(1-{p}_j\right)}{2}\kern1em \\ {}\kern1.5em \\ {}\kern2.5em =\kern1em {p}_i\kern0.5em -\kern0.5em {p_i}^2\kern0.5em +\kern0.5em {p}_j\kern0.5em -\kern0.5em {p_j}^2.\end{array} $$

Heterozygosity in an *i* * *j* cross is *H*_*ij*_ = *p*_*i*_(1 − *p*_*j*_) + (1 − *p*_*i*_)*p*_*j*_, and heterozygosity in an *s*_*i*_ * *j* cross is ${H}_{s_ij}={p}_{s_i}\left(1-{p}_j\right)+\left(1-{p}_{s_i}\right){p}_j$.

Thus, the heterozygosity excess of a cross relative to the mean of the pure lines is: ${H}_{ij}\kern0.5em -\kern0.5em {\overline{H}}_{ii,\;jj}={\left({p}_i-{p}_j\right)}^2$.

This result shows that, as expected [5], heterosis at the line level is proportional to the squared difference in allele frequency (SDAF) between the parental lines. It represents the between-line component of heterosis.

The heterozygosity excess of the offspring of *s*_*i*,*j*_ relative to the *i* * *j* cross is: ${H}_{s_ij}-{H}_{ij}=\left({p}_{s_i}-{p}_i\right)\left(1-2{p}_j\right)$.

This represents the within-line component of heterosis, and measures how much the expected performance of the offspring of this sire deviates from the mean of the cross, due to dominance. It is a combination of the deviation of the sire’s allele frequency from its line allele frequency, $\left({p}_{s_i}-{p}_i\right)$, and the dam-line allele frequency, (1 − 2*p*_*j*_).

Therefore, if we want to predict heterosis due to dominance for the offspring of an individual sire, then we need to sum the heterozygosity excess of the *i* * *j* cross relative to the mean of the two pure lines and the heterozygosity excess of the offspring of *s*_*i*,*j*_ relative to the *i* * *j* cross. Thus, the ${x}_{s_i,j}$ term in Equation 1 should be:

$$ {x}_{s_i,j}={\left({p}_i-{p}_j\right)}^2+\left({p}_{s_i}-{p}_i\right)\left(1-2{p}_j\right). $$

In the following text, we refer to ${x}_{s_i,j}$ as the “heterozygosity excess”.

We calculated the heterozygosity excess for the *s* = 1 to 3427 sires in our dataset and all dam-lines that they had been mated to. This was calculated for each SNP and then averaged across all SNPs. We used the sire allele frequencies $\left({p}_{s_i}\right)$, and missing SNPs were replaced by the sire’s line allele frequency at that SNP. Thus, the genome-wide average heterozygosity excess for offspring of sire *s*_*i*_ mated to dam line *j* was:

$$ {\overline{x}}_{s_{i,\;j}}=\frac{{\displaystyle \sum_{n=1}^N\left[{\left({p}_i-{p}_j\right)}^2+\left({p}_{s_i}-{p}_i\right)\left(1-2{p}_j\right)\right]}}{N}, $$

where *N* was the total number of SNPs.

#### Prediction of heterosis at the sire level

Following from the derivation above, we predicted the heterosis per sire by fitting a linear mixed model, where we regressed phenotypes of crossbreds on the genome-wide average heterozygosity excess, ${\overline{x}}_{s_{i,\;j}}$:

2 $$ \begin{array}{c}\hfill {y}_{s_i jklm}=\kern0.5em \mu + sire\_lin{e}_i + dam\_lin{e}_j+\beta \cdot {\overline{x}}_{s_{i,\;j}}+\hfill \\ {}\hfill tes{t}_k+\kern0.5em  hen\  densit{y}_{l:k}+HR{T}_m+\kern0.5em {e}_{ijklm}\hfill \end{array}, $$

where ${\mathrm{y}}_{{\mathrm{s}}_{\mathrm{i}}\mathrm{jklm}}$ is a phenotypic record, *sire_line*_*i*_ and *dam_line*_*j*_ are the fixed effects of the *i*^*th*^ sire-line and *j*^*th*^ dam-line of each cross (*i* = 1 to 4, *j* = 1 to 7), *β* is the partial regression coefficient of *y* on ${\overline{x}}_{s_{i,\;j}}$, *test*_*k*_ is the fixed effect of each performance test (*k* = 1 to 33 year-farm classes), *hen density*_*l*_ is a fixed effect accounting for the initial number of hens within a cage (*l* = 1 to 128); it was nested within *test* because the physical size of cages differed across some performance tests. The combined effect of the *H*en-house, *R*ow and *T*ier of the cage was accounted for by including the term “*HRT*_*m*_” as a random effect (*m* = 1 to 767). *e*_*ijklm*_ is the random residual error term. Data were analysed using the MIXED procedure in SAS version 9.2. This model was used for both traits.

For the crossbred offspring of each sire, predicted heterosis was calculated by multiplying the estimated regression coefficient of the phenotypes on ${\overline{x}}_{s_{i,\;j}}\kern0.24em \left({\widehat{\upbeta}}_{\mathrm{trait}}\right)$, by the ${\overline{x}}_{s_{i,\;j}}$ value between sire *s*_*i*_ and dam-line *j*:

3 $$ \mathrm{Predicted}\kern0.75em \mathrm{heterosi}{\mathrm{s}}_{\mathrm{trait},\ {\mathrm{s}}_{\mathrm{i}}\mathrm{j}}=\kern0.5em {\widehat{\beta}}_{trait}\cdot {\overline{x}}_{s_ij}\kern0.5em . $$

To determine the relative importance of using individual sire genotypes to predict heterosis at the sire level versus predicting heterosis only at the line level, we partitioned the heterozygosity excess into its between-line, (*p*_*i*_ − *p*_*j*_)^2^, and within-line, $\left(\left({p}_{s_i}-{p}_i\right)\left(1-2{p}_j\right)\right)$, components and calculated the variance explained by each. We also estimated regression coefficients of the phenotypes on the two components of heterozygosity excess, using the following model:

4 $$ \begin{array}{l}{y}_{s_i jklm}=\kern0.5em \mu + sire\_lin{e}_i + dam\_lin{e}_j\kern0.5em +\kern0.5em {\beta}_1\cdot {\left({p}_i-{p}_j\right)}^2+\\ {}\kern4em {\beta}_2\cdot \kern0.5em \left(\left({p}_{s_i}-{p}_i\right)\left(1-2{p}_j\right)\right)+tes{t}_k+\kern0.5em  hen\  densit{y}_{l:k}+HR{T}_m+\kern0.5em {e}_{ijklm}\\ {}\kern3.12em \end{array} $$

All model terms except *β*_1_ and *β*_2_ are the same as in Model 1 above. Also note that (*p*_*i*_ − *p*_*j*_)^2^ is the same as the squared difference in allele frequency (SDAF).

## Results and discussion

### Descriptive statistics

Table 1 shows the number of sires, records and mean values for egg number and weight for the 16 crosses in our study. Cage-based egg numbers ranged from 163.9 to 375.3. The S5*D3 cross had the highest mean egg number, i.e. 345.2, whereas the S4*D6 had the lowest mean egg number, i.e. 326.1. Cage-based egg weight ranged from 51.0 to 76.7 g. The mean egg weight was highest for the S5*D5 cross, i.e. 62.9 g and lowest for S1*D3, i.e. 59.9 g. Values of the genome-wide average heterozygosity excess, ${\overline{x}}_{s_{i,\;j}}$, ranged from 0.08 to 0.18, with an average of 0.12 and a standard deviation of 0.018.

#### Pure lines

The proportion of polymorphic SNPs was 0.37 for D1, 0.50 for S1, 0.42 for S4, 0.52 for S5, and 0.74 across all lines. From these polymorphic SNPs, expected heterozygosity was 0.314 for D1, 0.318 for S1, 0.288 for S4 and 0.296 for S5. The following heritabilities are averages of estimates for lines D1, S1, S4 and S5: heritability for egg production from 100 to 168 days of age was ~0.46 and that for egg production from 169 to 560 days of age was ~0.26. The heritability for egg weight over the entire production period was ~0.6.

### Predicted heterosis per sire

Using the hypothesis that heterosis is due to dominance, Amuzu-Aweh *et al.* [1] showed that by using the squared difference in allele frequency (SDAF) between parental pure lines, crossbred phenotypes can be partitioned into pure-line means and heterosis, even when pure-line phenotypes are unavailable. Here, we extended this concept by deriving the theoretical expectation for heterosis due to dominance expressed by the offspring of specific sires. We showed that the expected heterosis expressed by the offspring of a sire *s*_*i*_ from pure-line *i* mated to randomly chosen dams from pure-line *j* is a linear function of the heterozygosity excess in the offspring relative to mean heterozygosity of the pure lines.

Table 2 shows the estimated regression coefficients of egg number and egg weight on ${\overline{x}}_{s_{i,\;j}}$, along with their standard errors (se) and p-values. All fixed effects in the models were significant (p < 0.0001). The estimated regression coefficient of egg number on ${\overline{x}}_{s_i,j}$ was ${\widehat{\beta}}_{EN} = 93.5$ eggs and that of egg weight was ${\widehat{\beta}}_{EW} = 12.9\ \mathrm{g}$. The results in Table 2 show that there is a positive and highly significant association between ${\overline{x}}_{s_{i,\;j}}$ and crossbred performance for these traits, which indicates that the greater the heterozygosity excess is in the offspring of a particular sire, the higher the performance of its crossbred offspring is. Haberfeld *et al.* [6], who estimated correlations between heterosis and genetic distance between mating-pairs, concluded that offspring were superior when they were from mating-pairs with a relatively distant genetic relationship, but they compared sires from different lines. Our study shows that if heterosis is due to dominance, then also within a line, sires that are expected to produce offspring with higher heterosis when mated to the dam-line of interest can be identified and used for breeding.

**Table 2:** **Estimated regression coefficients of egg number and weight on heterozygosity excess** ^**1**^ **, their standard errors (se) and p-values**

	**Egg number**			**Egg weight (g)**		
	**Estimate**	**se**	**p-value**	**Estimate**	**se**	**p-value**
**Model 1**						
*β*	93.45	18.3	3.4 E-7	12.92	2.7	1.1 E-6
**Model 2**						
*β*_1_	92.5	19.3	2.2 E-6	12.94	2.8	4.7 E-7
*β*_2_	102.9	61.7	9.5 E-2	12.74	8.7	1.5 E-1

^1^ *β* is the partial regression coefficient of trait values on the full heterozygosity excess, ${\left({p}_i-{p}_j\right)}^2+\left({p}_{s_i}-{p}_i\right)\left(1-2{p}_j\right)$. *β* was estimated from Model 1; *β*_1_ is the partial regression coefficient of trait values on the between-line component (*p*_*i*_ − *p*_*j*_)^2^, and *β*_2_ is the partial regression coefficient of trait values on the within-line component, $\left({p}_{s_i}-{p}_i\right)\left(1-2{p}_j\right)$, of the heterozygosity excess. *β*_1_ and *β*_2_ were estimated simultaneously from Model 2.

**Figure 1:**
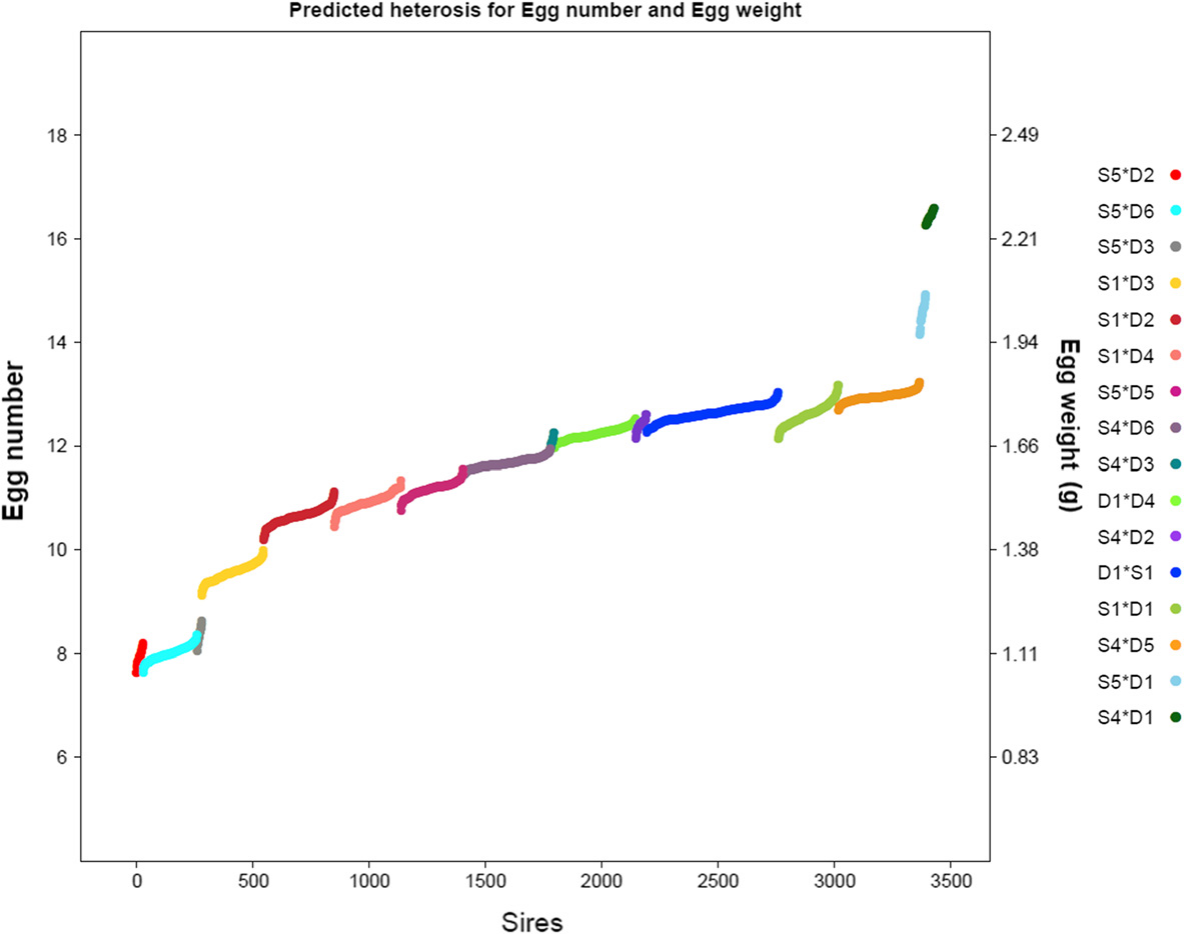
**Predicted heterosis in egg number and egg weight for the 3427 sires studied.** On the *x* axis, the sires are numbered from 1 to 3427 and the *y* axis shows predicted heterosis (left: egg number; right: egg weight (g)). Each point on the graph represents the average heterosis in the offspring of a particular sire; each sire was mated to one dam-line, but to several hens from that line. Colours represent the 16 crosses in this study.

Figure 1 shows the predicted heterosis for egg number and egg weight for the 3427 sires in our study. We predicted heterosis for both traits as the product of ${\widehat{\beta}}_{trait}$ and the heterozygosity excess between the sire and the dam-line (Equation 2). The heterozygosity excess for each sire*dam-line combination was the same for each trait. Thus, the predicted heterosis follows the same pattern for both traits, but is scaled by the value of ${\widehat{\beta}}_{trait}$. Predicted heterosis ranged from 7.6 to 16.7 for egg number, and from 1.1 to 2.3 g for egg weight. Predicted heterosis was lowest for an S5 sire mated to the D6 dam-line and highest for an S4 sire mated to the D1 dam-line. For both traits, the range of predicted heterosis was higher when prediction was done at the sire level than when it was done at the line level (line-level predictions not shown).

### Proportion of heterosis explained by the within-line sire variation

Next, we quantified the added value of using individual sire genotypes, rather than line allele frequencies, for the prediction of heterosis by comparing the variances of the within and between-line components of the heterozygosity excess. The total variance of ${\overline{x}}_{s_{i,\;j}}$ was 3.11E-4. The variance of the between-line component was 3.08E-4, and that of the within-line component was 0.0223E-4. Thus, the proportion of variance in ${\overline{x}}_{s_{i,\;j}}$ explained by the between-line component was 99.00%, and the proportion explained by the within-line component was 0.72% (the remaining 0.28% is due to a small positive covariance between the components). The extra genomic information from individual sires, therefore, explained only a small proportion of the total variance in heterozygosity excess, and thus a small proportion of the variance in predicted heterosis. This implies that most of the variation between sires is accounted for by line differences. Between lines, there was a difference of 9.1 in egg number and 1.3 g in egg weight for predicted heterosis for the offspring of the best and worst sire. Within lines, variation was greatest among the 318 S1 sires that were mated to the D2 dam-line: there was a difference of 1.0 in egg number and 0.14 g in egg weight between the offspring of the best and worst sires in this cross.

To further investigate the importance of the within-line component of the heterozygosity excess for prediction of heterosis, we fitted a model with a separate regression coefficient for each component of ${\overline{x}}_{s_{i,\;j}}$, for both egg number and egg weight (Model 2 in Methods section).

For both traits, the estimates of the two regression coefficients were very similar, but the regression coefficients on the within-line component of ${\overline{x}}_{s_{i,\;j}}$ were not statistically significantly different from zero (Table 2). The results suggest that the lack of statistical significance of ${\widehat{\beta}}_{2, trait}$ occurs because there was too little variation in the within-line component of ${\overline{x}}_{s_{i,\;j}}$, and thus too little power to accurately estimate *β*_2,*trait*_. The main reason for the low within-line variation in the heterozygosity excess is that we used an average over the entire genome, which reduces the within-line variance compared to that at the single SNP level. Given that ${\widehat{\beta}}_{2, trait}$ was not significantly different from zero, it is surprising that both regression coefficients were so similar, but this was probably due to chance. Hence, whether the within-line component of ${\overline{x}}_{s_{i,\;j}}$ would have good predictive ability for heterosis if there was enough within-line variation in ${\overline{x}}_{s_{i,\;j}}$ among sires cannot be evaluated based on this statistical analysis. However, it is important to note that when heterosis is entirely due dominance, *β*_1_ and *β*_2_ must have the same value.

In an analysis using only the between-line component of the heterozygosity excess, the standard errors of the estimated regression coefficients were slightly larger than when regressing on the full heterozygosity excess, ${\overline{x}}_{s_{i,\;j}}$. This shows that ${\widehat{\beta}}_{trait}$ was estimated more accurately when both the between- and within-line components of ${\overline{x}}_{s_{i,\;j}}$ were used. This also means that heterosis can be predicted more accurately when individual sire genotypes are used. Nonetheless, the 16 crosses in our study still ranked the same when either the full heterozygosity excess or only the between-line component was used as a predictor for heterosis, which indicates that both give corresponding predictions. Therefore, in a breeding program, the use of individual sire genotypes to predict heterosis may only be worthwhile if individual sire genotypes are already available as a result of routine genotyping.

### Model considerations

Another factor of interest is the level of linkage disequilibrium (LD) between the SNPs used and the loci that are relevant for heterosis/dominance. The essential assumption that underlies our approach is that genome-wide heterozygosity based on ~60K SNPs is a predictor of heterozygosity at the loci that affect the trait. Considering the proportion of polymorphic SNPs in each of the lines used in this study, we expect to have SNPs in LD with most, if not all, loci that are relevant to our target traits. In general, commercial White Leghorn laying hens have been found to have relatively high levels of LD [7,8], and SNP densities of 8 to 19K are considered to be sufficient for association mapping and implementation of genomic selection, provided that the SNPs are equally distributed across the genome in proportion to their recombination rates [8]. The SNPs used in this study meet these criteria [4].

Also, in our statistical model, we assumed that the dominance deviation at a locus is independent of the squared difference in allele frequencies between the parental lines at that locus. Note that this assumption does not require that SNPs are unlinked, or that SNPs are unlinked to QTL. It is unknown whether dominance effects at loci are correlated to allele frequency differences between lines. Selection for crossbred performance, however, could introduce such a correlation, since it may drive allele frequencies at loci with dominance in opposite directions in the two parental lines [9,10]. This would create a positive correlation between *d* and (*p*_*i*_ − *p*_*j*_)^2^. Moreover, our data do not represent a complete diallel cross, but a selected set of crosses, which are probably the crosses with above-average heterosis (most of these crosses had higher *predicted* heterosis than other potential crosses in the diallel set that were not made in practice [1]). Therefore, most crosses in this study are between lines that may have an above-average (*p*_*i*_ − *p*_*j*_)^2^ for loci showing dominance. This would also lead to a positive correlation between *d* and (*p*_*i*_ − *p*_*j*_)^2^. Such a positive correlation could result in biased estimation of *β*. With the present limited knowledge of the genome, however, we cannot quantify the effect of this bias on our estimates of *β*.

Furthermore, in our analyses, we used the average heterozygosity excess across the entire genome, which means that all SNPs were assumed to contribute equally to heterosis. An alternative would be to weight the SNPs based on their estimated contribution to heterosis, *i.e.* by their estimated dominance effect, *d*_*l*_. Dominance effects of SNPs can be estimated with, for example, single SNP regression models or with models that fit all SNPs simultaneously, such as those used for genomic selection (e.g. BayesD [11]). The relatively high accuracy with which between-line heterosis for egg number and egg weight can be predicted by averaging across the genome (See also [1]) suggests that heterosis is due to many loci with dominance effects, spread across the genome. This agrees with increasing evidence from genomic selection and genome-wide association studies that many traits in livestock are highly polygenic. The prediction of heterosis by weighting SNPs by their estimated dominance effects will be investigated in a future study.

## Conclusions

We derived an expression for the expected heterosis in the offspring of specific sires as the within- and between-line heterozygosity excess in the offspring of a sire and the dam-line that it is mated to, and used it to predict heterosis at the sire level.

We conclude that based on a dominance model, it is possible to predict heterosis for individual sires, and thus to identify sires with offspring that are expected to have relatively higher levels of heterosis than others. In our data, however, variation in predicted heterosis between sires within a line was small, and most differences in heterosis were observed between lines. We hypothesise that this method may work better if predictions are based on SNPs with identified dominance effects.
